# The impact of corporate digital transformation on the export product quality: Evidence from Chinese enterprises

**DOI:** 10.1371/journal.pone.0293461

**Published:** 2023-11-02

**Authors:** Jing Qian, Qunzhi She

**Affiliations:** School of Economics, Zhongnan University of Economics and Law, Wuhan, Hubei, China; Yunnan Technology and Business University, CHINA

## Abstract

The digital economy has become a driving force in the rapid development of the global economy and the promotion of export trade. Pivotal in its advent, the digital transformation of enterprises utilizes cloud computing, big data, artificial intelligence, and other digital technologies to provide an impetus for evolution and transformation in various industries and fields. in enhancing quality and efficiency. This has been critical for enhancing both quality and efficiency in enterprises based in the People’s Republic of China. Through the available data on its listed enterprises, this paper measures their digital transformation through a textual analysis and examines how this transformation influences their export product quality. We then explore the possible mechanisms at work in this influence from the perspective of enterprise heterogeneity. The results find that: (1) Digital transformation significantly enhances the export product quality in an enterprises, and the empirical findings still hold after a series of robustness tests; (2) Further mechanism analysis reveals that the digital transformation can positively affect export product quality through the two mechanisms of process productivity (*φ*), the ability to produce output using fewer variable inputs, and product productivity (*ξ*), the ability to produce quality with fewer fixed outlays; (3) In terms of enterprise heterogeneity, the impact of digital transformation on export product quality is significant for enterprises engaged in general trade or high-tech industries and those with strong corporate governance. In terms of heterogeneity in digital transformation of enterprise and the regional digital infrastructure level, the higher the level of digital transformation and regional digital infrastructure, the greater the impact of digital transformation on export product quality. This paper has practical implications for public policies that offer vital aid to enterprises as they seek digital transformation to remain sync with the digital economy, upgrade their product quality, and drive the sustainable, high-quality, and healthy development of their nation’s economy.

## Introduction

As the world’s leading exporter, China is accustomed to leveraging factor endowments like its abundance of labor and other comparative advantages as it engages in the international division of labor. The country’s export products tend to be low in price, low in quality, and high in quantity [1]. New trade theory (NTT) as represented by the enterprise heterogeneity model argues that a firm’s export product quality is directly related to its country’s export performance and the gains, status, and upgrading of its trade. Export product quality offers a new advantage for countries as they compete internationally [2–4]. The report of the 20th Congress of the Communist Party of China also states the necessity of “accelerating the building of a strong trade country.” At the same time, the existing scholarship indicates that while a series of initiatives have improved the quality of China’s export products, it remains relatively low overall [1],and the phrase “made in China” still represents a lower price and quality [5]. Improving the export product quality of Chinese enterprises will be vital in China’s construction of a large trade nation, high-quality economic development, and the transformation and upgrading of foreign trade.

The integration of digital technology and the real economy has formed a digital economy that is profoundly changing China’s traditional economic landscape as it reshapes the allocation of resources and the production activities of traditional enterprises. They country’s enterprises are also experiencing its multi-dimensional impact on their technological innovation, business management, and production methods [6]. The 14th Five-Year Plan (2021–2025) pledges to “develop the market for data factors, activate the potential of data factors, and drive changes in production, lifestyle and governance modes for digital transformation as a whole.” China’s government has proposed a series of policies to facilitate digital transformation, as it is well aware that the pace of the digital economy has made digitalization an inevitable choice for the transformation and reform of its enterprises [7, 8]. This transformation which has been called the most important strategic issue for businesses around the world is also inherently disruptive [9, 10] and can significantly affect the size, performance, and value added of a firm’s exports [11–13]. As such, will digital transformation aid or harm the export product quality of China’s enterprises, and how will it work?

To answer these questions, this paper explores the impact of digital transformation on the export product quality of enterprises from the macro background of the digital economy and the micro perspective of enterprises. Exploring this issue can enrich research on the economic effects of digital transformation in foreign trade and provide a theoretical basis for enhancing export product quality that reflects current circumstances. This is instrumental in both guiding the digital transformation of enterprises and sustaining the robust development of the Chinese economy. Finally, it serves as a valuable reference for developing countries as they seek to become more competitive in trade.

## Literature review

Present research on digital transformation and export product quality has focused on the factors that influence export product quality, the economic effects of digital transformation, and the impact of digital development on exports.

As for what factors influence export product quality in an enterprise, scholars have conducted studies either from the perspective of the market environment or internal factors within the enterprise. In terms of the market environment, factors such as FDI, OFDI, trade liberalization, minimum wage standards, government subsidies, industrial agglomeration, and the protection of intellectual property can all influence export product quality [14–23]. While minimum wage standards can harm export product quality [18], the other aforementioned factors can significantly enhance the export product quality. Among the internal factors within enterprises, those such as technological innovation, total factor productivity, input servitization, and enterprise listing have a significant positive impact on export product quality [24–27].

This paper also considers the literature on the economic effects of digital transformation in enterprises. The existing research defines digital transformation as the process of change in which firms use digital technologies to reduce the proportion of duplicated labor in production, operations, and services, or use advanced digital technologies to replace traditional ones [28]. Digital transformation can have a positive and beneficial impact in a variety of ways, including enhancing total factor productivity [29], fostering firm innovation [30], improving organizational efficiency [31], enhancing performance in capital markets [8], improving input-output efficiency [32], and promoting specialization and the division of labor [33].

With respect to the impact of digital development on enterprise exports, scholars argue that information technologies and the internet as the foundation of digital development, can reduce costs related to information, transactions, and risks in the process of trade [2, 34, 35] to form a new comparative advantage [36]. This can in turn boost exports [37–39] while improving their export performance [40], and moving them up the value chain [41, 42]. The information disclosure offered by information technology is one reason for an increase in quality [43]. Yi and Wang (2021) [12] find that digital transformation helps firms expand their exports, while Du *et al*. (2022) [44] hold that digital transformation has upgraded the quality of China’s export products by increasing innovation capacity, transforming products, and improving the quality of intermediate inputs. Hong et al. (2022) [45] make use of principal component analysis to measure the digital transformation indexes of firms, analyzing the U-shaped mediating role that innovation plays between digital transformation and export product quality.

In summary, the existing studies provide valuable reference information but are not without their limitations. First, while they confirm that digital technology can promote the upgrading of exports, they largely base their studies on the digital economy at the national and provincial levels [46]. Second, the existing literature at the micro level has studied the link between digital transformation and export trade but has yet to offer any theories as to its inner workings. This paper investigates the impact of digital transformation on the export product quality of enterprises and how this takes place from the perspective of the digital economy. Its marginal contributions are as follows: First, it explores the impact of digital transformation on firms at the micro level, which helps to enrich existing scholarship on the digital transformation of firms and international trade. Second, it examines precisely how digital transformation affects export product quality from the perspective of enterprise heterogeneity, helping to provide firm-level evidence for the contributions of the digital economy in China’s endeavors to cultivate a large trade nation.

## Theoretical mechanisms and research hypotheses

The advent of the digital economy has provided new avenues for enterprises diverse in scale to achieve success in export trade. Concurrently, digital transformation within firms has emerged as a crucial impetus for the advancement of export product quality. This paper amalgamates concepts from existing pertinent analyses, delving primarily into both direct and indirect effects. It scrutinizes the precise means through which digital transformation affects export product quality while presenting complementary research hypotheses.

### The direct effects of digital transformation on export product quality

With the rapid development of the digital economy, digital innovation and the application of big data have subverted the business management models of traditional enterprises and compelled them to transition into digital management [47]. However, digital transformation is not simply the combination and application of digital technologies. Digital transformation regards data as an equally important factor of production alongside labor and capital [48]. On the one hand, export enterprises strengthen their real-time monitoring and supervision of production processes by applying big data, cloud computing, and other digital technologies. While endeavoring to supervise the quality of their export products, enterprises continuously elevate and enhance their production processes, driving the development of products toward mechanization, intelligence, and automation [49, 50] to optimize product quality even further. On the other hand, export enterprises can avail themselves of big data to rapidly analyze and apprehend the dynamics of the international market and synchronize with demand in their target market. As they optimize and upgrade their products in response to customer feedback, they also manage to keep pace with demand in the international market. Simultaneously, the application of cross-border e-commerce, the Internet of Things, and other digital technologies have each condensed the landscape of international trade, optimized the exchange of information between buyers and sellers, reduced trade costs, and galvanized enterprises to participate in the export trade. It has also led to increasingly fierce competition in export markets and stimulated enterprises to continuously innovate and improve their product quality. Based on the above analysis, this paper proposes the following first hypothesis:

**H1**: Digital transformation can improve the export product quality of an enterprise.

### The indirect effects of digital transformation on export product quality

In this section, we draw on the theoretical framework of Hallak and Sivadasan (2013) [3] and Shi and Shao (2014) [25] to discuss the endogenous determinants of export product quality, and then analyze the theoretical mechanisms by which digital transformation affects the export product quality of enterprises.

### Analysis of the endogenous determinants of export product quality

Assuming that the consumer’s utility function is one of constant elasticity of substitution (CES), the function is as follows:

$$U={\left\{\int_{j\in \Omega }\;{\left(\lambda_{j}q_{j}\right)}^{\frac{\sigma -1}{\sigma }}dj\right\}}^{\frac{\sigma }{\sigma -1}}$$ (1)

Where *λ*_*j*_ is the quality of product *j* and *q*_*j*_ is the demand for product *j*, Ω denotes the mix of goods purchased by consumers, *σ* denotes the elasticity of substitution between products, and *σ*>1.

The price index corresponding to the above utility function is $P=\int_{j\in \Omega }\;p_{j}^{1-\sigma }\lambda_{j}^{\sigma -1}dj$. We can then obtain the demand equation for product *j*:

$$q_{j}=p_{j}^{-\sigma }\lambda_{j}^{\sigma -1}\frac{E}{P}$$ (2)

Where *E* is the total consumer expenditure, $\frac{E}{P}$ denotes the size of market demand, and $p_{j}^{\sigma }$ denotes the price of product *j*. Eq (2) indicates that the demand for product *j* depends on its price ($p_{j}^{\sigma }$) and quality (*λ*_*j*_).

We then introduce the production behavior of enterprises into the model. Based on the enterprise productivity heterogeneity of Meltiz [4], Hallak and Sivadasan (2013) [3] introduce two heterogeneous attributes into model: "process productivity" (*φ*) is the ability to produce output using fewer variable input; "product productivity” (*ξ*) is the ability to produce quality with fewer fixed outlays. These two heterogeneous attributes affect variable costs and fixed costs of enterprises, respectively, which in turn affect product quality. The variable costs (C) and fixed costs (F) specifically expressed as Eqs (3) and (4):

$$C=\frac{k}{\varphi }\lambda^{\beta }$$ (3)

$$F=F_{0}+\frac{f}{\xi }\lambda^{\alpha }$$ (4)

Where *β* denotes the quality elasticity of variable production costs, *α* denotes the quality elasticity of fixed costs, and 0<*β*<1, *α*>0. *φ* is the process productivity that represents the difference in variable costs between enterprises, and *ξ* is product productivity, representing the heterogeneity of quality production capability, it reflects the diverse fixed input efficiencies of enterprises, namely their ability to improve product quality under given fixed expenditures [3].

Given the demand and cost functions, the profit function of the enterprise is obtained as follows:

$$\pi =\frac{1}{\sigma }{\left(\frac{\lambda_{j}}{p_{j}}\right)}^{\sigma -1}\frac{E}{P}-F_{j}-f_{x}$$ (5)

Where *f*_*x*_ is the fixed trade cost. Using the firm’s profit-maximizing condition, its optimal product quality can be obtained as follows:

$$\lambda ={\left[\frac{1-\beta }{\alpha }{\left(\frac{\sigma -1}{\sigma }\right)}^{\sigma }{\left(\frac{\varphi }{\kappa }\right)}^{\sigma -1}\frac{\xi }{f}\frac{E}{P}\right]}^{\frac{1}{\alpha^{'}}}$$ (6)

Where *α*′ = *α*−(1−*β*)(*σ*−1)>0.

According to Eq (6), the optimal product quality of an enterprise is endogenously determined by the firm’s process productivity (*φ*) and product productivity (*ξ*). By taking the partial derivatives of Eq (6) with respect to *φ* and *ξ*, we can obtain $\frac{d\lambda }{d\varphi }>0,\;\frac{d\lambda }{d\xi }>0$. This indicates that the higher the firm’s process productivity and product productivity, the higher the quality of its products. In other words, an enterprise can improve its export product quality by increasing its process productivity (*φ*) and product productivity (*ξ*).

### Mechanism analysis of how digital transformation influences export product quality

Based on the above analysis of the endogenous determinants of export product quality, it is evident that process productivity (*φ*) and product productivity (*ξ*) are its two major determinants. In this section, we will further investigate the mechanisms through which digital transformation affects export product quality from the perspectives of these two factors.

### Process productivity (*φ*)

Digital transformation offers a comprehensive optimization and upgrading of production in an enterprise. The application of digital technologies can directly reduce the waste of production factors, enhance the unit factor output of enterprises, and invigorate overall process productivity [51]. Simultaneously, the application of digital technologies such as artificial intelligence and big data ensures the efficient transmission of information among departments within enterprises which reduces information asymmetry, saves on various costs in the production process, and augments production efficiency [2]. The above analysis indicates that digital transformation can either directly or indirectly improve a firm’s process productivity *φ*, i.e., $\frac{d\varphi }{ddig}>0$ (where dig denotes the digital transformation of an enterprise). According to Eq (6), it can be inferred that the higher the process productivity of a firm, the higher the quality of its products, $\frac{d\lambda }{d\varphi }>0$. This according to the chain rule is $\frac{d\lambda }{ddig}=\frac{d\lambda }{d\varphi }\cdot \frac{d\varphi }{ddig}>0$. This is to say that the digital transformation of an enterprise can elevate its export product quality by increasing its process productivity. Accordingly, this paper proposes the following second hypothesis:

**H2**: Digital transformation can improve the export product quality of an enterprise by improving its process productivity.

### Product productivity (*ξ*)

Product productivity *ξ* primarily reflects the ability of a firm to improve product quality under given fixed expenditures [25]. However, the enhancement of product quality in an enterprises is closely associated with the innovation that is accomplished through research and development (R&D) activities [2, 25, 52, 53]. The existing research indicates that the continuous integration of digital technology and the real economy stimulates enterprises to increase investment in R&D and innovation [54], which contributes to enhancing a firm’s product productivity (*ξ*) [21, 55]. First, the inherent innovative elements of digital products are assimilated into an enterprise when they are incorporated as a factor input in the production process. This enables enterprises to efficiently absorb external knowledge, reduce transaction costs, enhance their R&D capabilities, and promote the continuous optimization and refinement of their products, thereby enhancing export product quality. Second, the digital industry itself is highly capable of the efficient transmission of information and real-time feedback mechanisms. Digital transformation assists enterprises in gaining an in-depth and timely understanding of the production process and market responses of their products [21]. This enables enterprises to promptly identify the strengths and weaknesses of their products, thereby motivating them to engage in product upgrades and innovation activities to meet the demands of consumers and ultimately enhance their product productivity. In conclusion, digital transformation can to some extent facilitate the enhancement of product productivity, i.e., $\frac{d\xi }{ddig}>0$. As indicated by Eq (6), the higher the product productivity of a firm, the higher the quality of the products it produces, i.e., $\frac{d\lambda }{d\xi }>0$. According to the chain rule, $\frac{d\lambda }{ddig}=\frac{d\lambda }{d\xi }\cdot \frac{d\xi }{ddig}>0$. This suggests that digital transformation can drive the improvement of export product quality through the mechanism of product productivity. This paper therefore proposes a third hypothesis:

**H3**: Digital transformation can positively impact export product quality by improving its product productivity.

## Study design

### Selection and measurement of variables

#### Selection and measurement of export product quality

This paper refers to Khandelwal [56], Shi and Shao [25], and Xu and Wang [18] to measure the export product quality. We estimate China’s export product quality in the four dimensions of the firm, product, importing country, and year using the demand information regression inference method. The quantity of product *j* exported by enterprise *i* to country *m* in year *t* is:

$$q_{ijmt}={\lambda_{ijmt}}^{\sigma -1}{p_{ijmt}}^{-\sigma }\left(\frac{E_{mt}}{P_{mt}}\right)$$ (7)

Where *λ*_*ijmt*_ is the quality of product *j* exported by enterprise *i* to country *m* in year *t*, *σ* is the elasticity of substitution for the product type, *p*_*mt*_ is the price index of the importing country, and *E*_*mt*_ is the market size.

Taking the natural logarithm of both sides of Eq (7), we can obtain that:

$${lnq}_{ijmt}=\chi_{mt}-\sigma lnp_{ijmt}+E_{ijmt}$$ (8)

where *χ*_*mt*_ = *lnE*_*mt*_−*lnP*_*mt*_ is the importing country-year dummy variable to control for variables such as import distance that vary only with the importing country, variables such as exchange rates that vary only with time, and variables such as GDP that vary with time and importing country. ℰ_*ijmt*_ = (*σ*−1)*lnλ*_*ijmt*_ is a residual term that contains information on product quality. Considering that quality *q*_*ijmt*_ is related to price *p*_*ijmt*_ and this can lead to problems of endogeneity, this paper draws on the study of Shi and Shao [25] by choosing the average price of a firm’s exported products in other markets as the instrumental variable for the price of its exported products in country *m*.

After considering endogeneity problems, this paper regresses Eq (8). According to the results, we can obtain the exported product quality as follows:

$${quality}_{ijmt}=ln\hat{\lambda_{ijmt}}=\frac{\hat{E_{ijmt}}}{\sigma -1}=\frac{{lnq}_{ijmt}-ln\hat{q_{ijmt}}}{\sigma -1}$$ (9)

Where *quality*_*ijmt*_ is the quality of product *j* exported by enterprise *i* to country *m* in year *t*. We further standardize the product quality of Eq (9) for the following result:

$${r_{quality}}_{ijmt}=\frac{{quality}_{ijmt}-{minquality}_{ijmt}}{{maxquality}_{ijmt}-{minquality}_{ijmt}}$$ (10)

Where *maxquality*_*ijmt*_ denotes the maximum values of product quality for a given product *j* at the level of all years, all enterprises, and all importing countries. *minquality*_*ijmt*_ denotes the minimum values of product quality for a given product *j* at the level of all years, all enterprises, and all importing countries. The standardized product quality is in the range of 0 to 1.

### Selection and measurement of digital transformation in an enterprise

This paper draws on the latest research [8, 29, 33] to measure the degree of digital transformation in an enterprise. First, we refer to the work of Wu et al. (2021) [8] to establish a relatively complete corpus for digitization. The specific corpus used to analyze the frequency of digitization-related words is shown in Table 1. Second, we use python software to analyze the *Management Discussion and Analysis* (MD&A) section of the annual reports of listed companies by extracting the frequency of digitization-related words. Finally, the frequency of these words in the annual reports of listed companies (+1 is taken as a logarithm) is used as an indicator to measure the degree of digital transformation in an enterprise.

**Table 1:** Digitization dictionary.

Core Vocabulary	High Frequency Vocabulary	Underlying vocabulary
Artificial Intelligence	Artificial intelligence (4) Intelligent robots (16) Face recognition (17)	Machine learning, brain-like computing, artificial intelligence, face recognition, cognitive computing, business intelligence, identity verification, deep learning, bioidentification technology, image understanding, semantic search, intelligent data analysis, natural language processing, intelligent robotics
Blockchain	Digital Currency (39)	Bitcoin, alliance chain, decentralization, distributed computing, digital currency
Cloud Computing	Internet of things (1) Cloud computing (3)	EB-level storage, multi-party secure computing, consensus mechanisms, stream computing, green computing, in-memory computing, converged architectures, data mining, graph computing, Internet of Things, information physical systems, cloud computing
Big Data	Electronic credit (14) Virtual reality (20)	Mixed reality, text mining, data visualization, virtual reality, billion concurrency, heterogeneous data, augmented reality, E-collection
Digital Technology Usage	E-commerce (2) Mobile Internet (5) Internet finance (6) Smart grid (7) Industrial Internet (8) Financial technology (9) Intelligent transportation (10) Smart home (11) Digital marketing (12) Mobile Internet (13) Mobile payment (14) Third-party payment (18) Autonomous driving (19)	B2B, B2C, C2B, C2C, Fintech, NFC, e-payment, O2O, third-party payment, e-commerce, industrial Internet, Internet finance, Internet healthcare, financial technology, open banking, quantitative finance, digital finance, digital marketing, netlink, unmanned retail, mobile Internet, mobile Internet, mobile payment, voice recognition, smart agriculture, smart wear, smart grid, smart contract, smart environmental protection, smart home, smart transportation, smart customer service, smart energy, smart investment, smart travel, smart medical, smart marketing, autonomous driving

Note: The ranking of vocabulary word frequency is in parentheses

Artificial intelligence, blockchains, cloud computing, and big data form the basis of the usage of digital technology in Table 1. The underlying vocabulary is seen as the statistical vocabulary of specific digital word frequencies, and high-frequency vocabulary is considered to be the number of words with the highest number of occurrences among all words. It is apparent that the terms *artificial intelligence*, *digital currency*, *the Internet of Things*, *electronic credit*, and *e-commerce* have the highest frequency under *artificial intelligence*, *blockchains*, *cloud computing*, *big data*, and *digital technology usage*.

**Fig 1:**
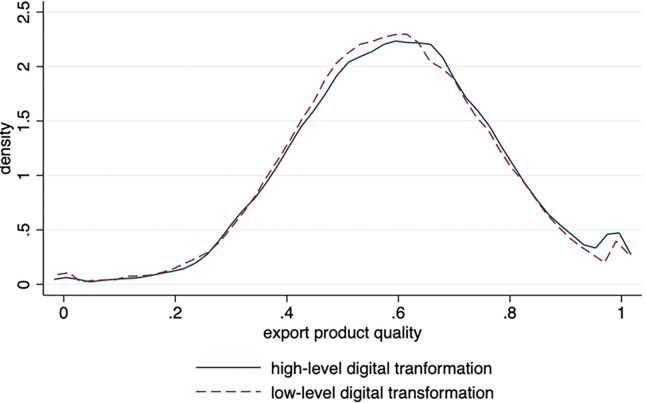
Kernel density map of export product quality for digital transformation.

In accordance with the results measured from export product quality indexes and the level of digital transformation among enterprises, this paper divides digital transformation into high and low categories according to the mean value for typical factual analysis. Fig 1 indicates that the kernel density curve for the export product quality of highly digitized enterprises falls more to the right than those with low digitization. This means that highly digitized enterprises also have a higher export product quality. Hypothesis 1 can be preliminarily verified, as the export product quality of enterprises improves with their digital transformation.

### The selection and measurement of mechanism variables

Mechanism analysis suggests that the digital transformation of an enterprise can improve export product quality through the two mechanisms of process productivity (*φ*) and product productivity (*ξ*). Among these, process productivity (*φ*) is the ability to produce an output using fewer variable inputs and is measured by total factor productivity. Considering the availability and completeness of the data, this study employs the Levinsohn-Petrin (LP) and Fixed Effects (FE) methods to estimate total factor productivity in reference to Chen [57]. Another mechanism variable is product productivity (*ξ*), which is the ability to produce quality with fewer fixed outlays. The existing databases lack the information needed to directly ascertain product productivity (*ξ*), however. Considering that R&D expenditure is avital component of fixed expenditures and higher R&D efficiency is associated with stronger product productivity, there exists a strong positive correlation between the two [4, 25, 52]. Therefore, enhancing the product productivity of a firm requires continuous R&D and innovation activities to improve its product quality. This paper measures product productivity (*ξ*) according to the quantity of patent innovation (R&D_q) and innovation efficiency (R&D_e), and the specific calculating methods with reference to He *et al*. [58] are as follows:

$$R\&D\_q=ln\;({AppPatent}_{i,t}+{GranPatent}_{i,t})$$

$$R\&D\_e=ln\;(1+{AppPatent}_{i,t})/ln\;(1+{Research}_{i,t})$$

Where *AppPatent*_*i*,*t*_ represents enterprise *i*’s quantity of patent applications in year *t*, *GranPatent*_*i*,*t*_ represents enterprise *i*’s quantity of granted patents in year *t* and *Research*_*i*,*t*_ represents enterprise *i*’s total R&D investment in year *t*.

### The selection and measurement of control variables

This paper selects a series of control variables that can impact the export product quality of an enterprise with reference to relevant studies [1, 38, 59–61]: *enterprise scale (lnes*), described by the natural logarithm of the firm’s total annual assets; *the rate of return on total assets (rrt)*,represented by the net profit of the firm divided by the average balance of total assets; *management fee rate (mfr)*, measured by dividing the firm’s management fee by its operating revenue measures; *Tobin’s Q (tq)*, which measures a firm’s performance and growth by dividing the sum of the firm’s total market value and total liabilities by its total assets; and *government subsidies (lngs)*, which in this paper is represented by the amount of government subsidies received by an enterprise. The descriptive statistics of specific variables are shown in Table 2.

**Table 2:** Summary statistics for variables.

Variable	Obs	Mean	Std. Dev.	Min	Max
Export product quality	111345	0.59	0.175	0	1
Digital transformation	111345	3.696	0.295	1.927	5.331
Enterprise scale	111345	21.992	1.342	16.412	27.569
rate of return on total assets	111345	0.05	0.078	-9.117	20.788
management fee rate	111345	0.085	2.046	-8.41	1066.288
Tobin’s Q	103183	0.555	0.424	-0.367	1.234
government subsidies	101221	16.382	1.788	2.536	22.2

### Data sources

The data concerning export product quality in this paper were obtained from the China Customs Database. This paper refers to Shi and Shao’s [25] method of data processing to exclude the samples of enterprises with incomplete information and unreasonable data (e.g., export amounts under 50 or export quantities less than 100), the samples of enterprises with intermediate traders (enterprises with names like *trade*, *commerce*, *science and trade*, *economic and trade*, *import and export* are considered as such). The elasticity of substitution between products must be calculated artificially, and this paper uses the method of Broda and Weinstein [62].

The financial data of listed companies used in this paper were obtained from databases such as CSMAR, INCOPAT, and CNRDS. Data regarding digital transformation were obtained from the annual reports of each company.

After matching the data concerning a firm’s export product quality with the financial data of listed enterprises, this created a total study sample of 4,403 listed enterprises containing 111,345 observations at the firm-product-importing country-year level for the period of 2000 to 2015. With reference to common practices in the existing literature, the data were screened, and the following were excluded: (1) samples with serious missing core variables; (2) ST and *ST samples; and (3) samples of financial firms.

### Model setting

The following benchmark model was constructed to examine the impact of digital transformation on the export product quality of enterprises:

$${\mathrm{quality}}_{ijmt}=\alpha +\beta {\mathrm{Dig}}_{it}+\sum \mathrm{Controls}+{Year}_{t}+{Indus}_{i}+u_{ijmt}$$ (11)

Where subscript *i* represents different enterprises, *j* represents different products, *m* represents importing countries, and *t* represents time; *quality*_*ijmt*_ denotes the quality of product *j* exported by enterprise *i* to country *m* in year *t*, *Dig* denotes the degree of digital transformation of enterprise *i* in year *t*, and *Controls* is the control variable group that specifically contains enterprise scale, the rate of return on total assets, the management fee rate, Tobin’s Q, and government subsidies. *Year*_*t*_ is the year-fixed effect, *Indus*_*i*_ is the industry-fixed effect, and *u*_*ijmt*_ is the random disturbance term. This paper draws on the studies of Zhou [63] and DeHaan E [64] to analyse industry fixed effects in comparison with firm fixed effects, and the results suggest that industry fixed effects are superior to firm fixed effects.

## Empirical analysis

### Baseline analysis

This paper examines the impact of digital transformation on the export product quality of firms, and the specific test results are displayed in Table 3.

**Table 3:** Benchmark regression.

Variable	(1)	(2)	(3)	(4)
Dig	0.017***	0.006*	0.021***	0.007*
	(0.003)	(0.004)	(0.003)	(0.004)
lnes	0.005***	0.004***	0.004***	0.003***
	(0.001)	(0.001)	(0.001)	(0.001)
rrt	-0.055***	-0.043***	-0.040***	-0.021**
	(0.010)	(0.010)	(0.011)	(0.011)
mfr	-0.055***	-0.096***	0.054***	0.004
	(0.012)	(0.013)	(0.014)	(0.014)
tq	0.020***	0.016***	0.027***	0.023***
	(0.002)	(0.002)	(0.002)	(0.002)
gs	-0.006***	-0.006***	-0.005***	-0.005***
	(0.001)	(0.001)	(0.001)	(0.001)
Constant	0.535***	0.607***	0.511***	0.602***
	(0.015)	(0.016)	(0.017)	(0.019)
Year effect	No	Yes	No	Yes
Industry effect	No	No	Yes	Yes
Observations	101221	101221	101221	101221
R-squared	0.004	0.011	0.028	0.034

Notes: The t-statistic in parenthesis.

***, **, and * indicate statistically significant at 1%, 5%, and 10%, respectively.

Table 3 presents the baseline regression results for the benchmark model (Eq 11). The results in column (1) of Table 3 indicate that the digital transformation of a firm can indeed improve its export product quality when year-fixed and industry-fixed effects are not considered. The results in columns (2) and (3) of Table 3 demonstrate that digital transformation still has a significant positive impact on export product quality even when these two effects are considered. The results in column (4) of Table 3 show that the explanatory variable coefficients are still significantly positive, which indicates that the digital transformation of enterprises can improve export product quality when year-fixed and industry-fixed effects are both considered in the model. This is consistent both with the above theoretical analysis of direct effects and the findings of similar existing studies [45], Hereby proving Hypothesis 1.

### Robustness test

To further confirm that the results of the benchmark regression are robust and credible, this paper conducts robustness tests in five aspects: replacing explanatory variables, adjusting export product quality measures, considering regional effect, eliminating the impact of annual variation in industry product quality, and considering endogeneity.

### Replacing explanatory variables

The explanatory variables in this paper are expressed through the frequency of specific words that relate to digitization in the statements of firms. This method of measurement does not consider a firm’s investment in digital transformation, however. This paper therefore draws on the study of Song et al. [7] to measure digital transformation by the proportion of total intangible assets that are related to digitization. The test results are displayed in column (1) of Table 4.

**Table 4:** Results of robustness tests.

Variable	(1)	(2)	(3)	(4)	(5)	(6)	(7)	(8)	(9)	(10)
	quality	A-quality	quality	quality	quality	quality	quality	quality	quality	quality
Intangible assets	0.101***									
	(0.018)									
Dig		0.069***	0.013***	0.022***	0.011***	0.016***	0.025***	0.415***	0.017**	0.016*
		(0.003)	(0.004)	(0.004)	(0.004)	(0.004)	(0.005)	(0.082)	(0.008)	(0.008)
lnes	0.007***	-0.009***	0.006***	0.005***	0.006***	0.008***	0.008***	0.009***	-0.009*	-0.007
	(0.001)	(0.001)	(0.001)	(0.001)	(0.001)	(0.001)	(0.001)	(0.002)	(0.005)	(0.005)
rrt	-0.052***	0.119***	-0.063***	-0.024	-0.018	-0.072***	-0.009	-0.008	0.092**	0.082**
	(0.010)	(0.008)	(0.012)	(0.015)	(0.011)	(0.013)	(0.016)	(0.012)	(0.037)	(0.036)
mfr	-0.043***	-0.008	-0.014	0.031*	0.032**	0.002	0.046***	-0.081***	0.049	0.058
	(0.015)	(0.011)	(0.015)	(0.016)	(0.014)	(0.015)	(0.017)	(0.023)	(0.046)	(0.046)
tq	0.001	-0.012***	0.016***	0.006**	0.022***	0.017***	0.010***	0.050***	0.019***	0.016**
	(0.002)	(0.001)	(0.002)	(0.002)	(0.002)	(0.002)	(0.002)	(0.006)	(0.007)	(0.007)
lngs	-0.005***	-0.004***	-0.005***	-0.007***	-0.008***	-0.007***	-0.010***	-0.012***	0.001	0.001
	(0.001)	(0.000)	(0.001)	(0.001)	(0.001)	(0.001)	(0.001)	(0.001)	(0.001)	(0.001)
Constant	0.530***	0.343***	0.516***	0.525***	0.559***	0.497***	0.510***		0.722***	0.669***
	(0.017)	(0.015)	(0.021)	(0.025)	(0.021)	(0.022)	(0.026)		(0.108)	(0.107)
Year effect	Yes	Yes	Yes	Yes	Yes	Yes	Yes	Yes	Yes	Yes
Industry effect	Yes	Yes	Yes	Yes	Yes			Yes	Yes	Yes
Province effect			Yes			Yes				
City effect				Yes			Yes			
Year*Ind					Yes	Yes	Yes			
Country effect									Yes	Yes
Year*Ind* Country									Yes	Yes
Id effect									Yes	Yes
Trade effect										Yes
First stage F value								49.02		
Wald test								253.351***		
Observations	127350	92505	101221	101221	101220	101220	101220	97260	98594	98593
R-squared	0.033	0.276	0.041	0.061	0.044	0.051	0.068	0.104	0.261	0.270

Notes: The t-statistic in parenthesis.

***, **, and * indicate statistically significant at 1%, 5%, and 10%, respectively.

### Adjusting export product quality measures

The elasticity of substitution between products must be calculated artificially in the process of measuring export product quality. This paper uses the method of Broda and Weinstein to calculate the alternative elasticity between products in baseline regression. Following the approach of Fan and Guo (2015) [65], an alternative elasticity of σ = 5 is employed to recompute the export product quality (A-quality) for the conducting of robustness tests and ensure the robustness of the estimation results. The results of the tests are presented in column (2) of Table 4.

### Regional effects

Considering the variations in economic development and policy regulations across different provinces where firms are located, this study tests robustness by incorporating controls for provincial and industry influences. The results are presented in column (3) of Table 4. Additionally, this study further controls for city and industry effects due to the possibility of city-level characteristics remaining constant over time. The results are presented in column (4) of Table 4.

### Eliminating the impact of annual variations in industry product quality

Considering that trends in product quality may vary across different industries over time, it is possible for some industries to experience a decline in product quality and a larger proportion of poorly digitized firms while others see the reverse. While this study has already controlled for industry fixed effects in product quality, it is necessary to further account for annual changes in industry product quality. Therefore, this paper incorporates controls for year-industry interaction effects and regional effects to eliminate the impact of annual variations in industry product quality. The results of this analysis are presented in columns (5)-(7) of Table 4.

### Considering endogeneity

This study investigates how the digital transformation of enterprises enhances export product quality, and it is possible for a bi-directional causality to exist between digital transformation and the export product quality. Specifically, enterprises with a higher export product quality may also have a stronger willingness to adopt digital technologies. This bi-directional causality relationship may pose endogeneity challenges to the results of the benchmark model. Drawing on the relevant research [66], this paper employs the fiber optic cable length as an instrumental variable for digital transformation and uses the two-stage least square method for estimation. The primary reasons for selecting this instrumental variable are as follows: (1) Fiber optic cable length is the most vital aspect of digital infrastructure in the digital era [67]. The length of fiber-optic cables represents the capacity for the high-speed and stable transmission of digital information. The length demanded of fiber optic cable routes increases in tandem with the demand for larger network bandwidth as enterprises undergo digital transformation, and the fact that this length is so closely intertwined with the progress of this transformation thoroughly satisfies the relevance condition. (2) Since China’s fiber optic cables are primarily controlled and maintained by its four major state-owned network operators, this instrumental variable is unrelated to export product quality, satisfying the exogeneity assumption for the instrumental variable. The results of the endogeneity test in this paper are shown in column (8) of Table 4.

### Other robustness tests

This study also conducts a series of other robustness tests. Considering that factors such as the destination country and the trade mode adopted by the enterprise may also have an impact on the explained variable, we further control for these two effects to minimize any problems caused by the omission of important variables. We also consider that the export product quality of an enterprise may change over time, particularly in terms of trends like increasing product standards in the destination country. We therefore further control the interaction effects of *time-industry-export destination country* to comprehensively address issues concerning omitted variables. The results are shown in columns (9) and (10) of Table 4.

The results in Table 4 reveal that the explanatory variable coefficients are significantly positive after a series of robustness tests, offering evidence that the primary findings in this paper are indeed robust.

### Further analysis

#### Mechanism analysis

After empirically verifying the effect of digital transformation on export product quality, we further test their underlying influence mechanisms in this section. Based on the above theoretical analysis, the impact of digital transformation on export product quality can be realized through the two mechanisms of process productivity (*φ*) and product productivity (*ξ*).

To further ascertain whether process productivity (*φ*) and product productivity (*ξ*) present a mediation effect between digital transformation and quality, this paper draws on the logic of mechanism analysis [68–72], establishing Eq (12) to test the mechanisms by which the digital transformation of an enterprise improves its export product quality.

$$M=\alpha +\beta {\mathrm{Dig}}_{j,t}+\sum {\mathrm{Controls}}_{i,t}+Year+Indus+u_{i,t}$$ (12)

Where *M* denotes the variable of the mechanism and the meaning of the remaining variables is consistent with Eq (11). Considering the problem of endogeneity between the mechanism variables and explanatory variable, the two-stage least square method is used to empirically test the impact of digital transformation on mechanism variables. This paper refers to recent research [73, 74] in selecting the number of internet broadband access ports and domain names as the instrumental variable, the primary reasons for which are as follows: (1) internet broadband access ports and domain names are two crucial elements in the integration of the real and digital economies, and intuitively reflect the maturation of digital transformation, which satisfies the relevance condition; (2) internet broadband access ports and domain names are not directly correlated with export product quality, which satisfies the assumption of exogeneity in the selection of instrumental variables. The specific test results are displayed in Table 5.

**Table 5:** Results of mechanism test.

Variables	(1)	(2)	(3)	(4)
	tfp_lp	tfp_fe	R&D_q	R&D_e
Dig	0.160***	0.081***	0.573***	0.003*
	(0.007)	(0.007)	(0.025)	(0.001)
lnes	-0.669***	-0.866***	0.537***	0.016***
	(0.002)	(0.002)	(0.005)	(0.000)
rrt	-2.138***	-1.671***	1.273***	0.079***
	(0.020)	(0.020)	(0.065)	(0.003)
mfr	2.416***	2.673***	-0.000	-0.000
	(0.022)	(0.022)	(0.001)	(0.000)
tq	0.006*	-0.022***	-0.028**	-0.007***
	(0.004)	(0.004)	(0.012)	(0.001)
lngs	-0.008***	-0.019***	0.218***	0.009***
	(0.001)	(0.001)	(0.003)	(0.000)
Constant	23.597***	30.471***	-15.464***	-0.416***
	(0.038)	(0.037)	(0.122)	(0.007)
First stage F value	1.1e+05	1.3e+05	3924.72	3327.01
Wald test	127.986	127.986	166.819	119.902
Year effect	Yes	Yes	Yes	Yes
Industry effect	Yes	Yes	Yes	Yes
Observations	107459	107459	108333	98371
R-squared	0.905	0.943	0.597	0.434

Notes: The t-statistic in parenthesis.

***, **, and * indicate statistically significant at 1%, 5%, and 10%, respectively.

The results In columns (1) and (2) of Table 5 show that the coefficients of the explanatory variable (Dig) are significantly positive, indicating that the digital transformation of an enterprise significantly improves its process productivity (*φ*). The results in columns (3) and (4) of Table 5 show that the coefficients of the explanatory variable (Dig) are significantly positive, which indicates that the digital transformation of an enterprise also has a significant positive impact on its product productivity (*ξ*). Numerous studies have also illustrated the pivotal role played by process productivity (*φ*) and product productivity (*ξ*) in improving export product quality [25, 52, 53, 75–79]. With reference to these works, we can draw the conclusion that digital transformation can indeed influence the export product quality of an enterprise for the better through the two mechanisms of process productivity (*φ*) and product productivity (*ξ*), proving Hypotheses 2 and 3.

#### Heterogeneity analysis

*Enterprise heterogeneity*. Considering that the heterogeneity of an enterprise is an integral component of its export product quality [1], it is reasonable to take this into account when studying how it this affected by digital transformation. We define enterprise heterogeneity in this study in terms of a firm’s trade mode, corporate governance, and the industry to which it belongs. We then analyze this heterogeneity through a sub-sample test. First, digital transformation has different effects on the improvement of product quality for different trade modes. To test heterogeneity, the total sample in this study is divided into two sub-samples of general and processing trade modes. Second, firms with different levels of corporate governance have different requirements concerning their management, leading to different applications and absorption capacities for digital technology and different levels of production and operation. Digital transformation can therefore affect the improvement of product quality in a variety of ways. This paper draws on the study of Zhou et al. [80] to test heterogeneity by dividing the sample of enterprises into strong and weak governance categories. Finally, products with different technical attributes contain different technologies, and digital transformation will improve product quality differently depending on the technical level of the industry to which the enterprise belongs. Drawing on the research of Brockman et al. [81], this study also tests heterogeneity by categorizing industries as either high-tech or low-tech intensive based on the median intensity of R&D in each industry. The specific test results are shown in Table 6.

**Table 6:** Results of heterogeneity analysis.

Variables	(1)	(2)	(3)	(4)	(5)	(6)
	General trade	Processing trade	Strong governance	Weak governance	High-tech industries	Low-tech industries
Dig	0.013***	-0.002	0.014**	0.001	0.023***	-0.001
	(0.004)	(0.009)	(0.006)	(0.005)	(0.005)	(0.006)
lnes	0.003**	0.005***	0.003**	0.002	0.007***	0.002
	(0.001)	(0.002)	(0.001)	(0.001)	(0.001)	(0.002)
rrs	-0.018	-0.015	-0.011	0.003	0.037***	-0.133***
	(0.012)	(0.028)	(0.015)	(0.017)	(0.013)	(0.020)
mfr	0.032**	-0.079**	0.095***	-0.131***	0.134***	-0.245***
	(0.015)	(0.038)	(0.017)	(0.025)	(0.016)	(0.026)
tq	0.029***	0.002	0.026***	0.009***	0.025***	0.028***
	(0.002)	(0.004)	(0.003)	(0.003)	(0.002)	(0.003)
lngs	-0.005***	-0.006***	-0.005***	-0.006***	-0.009***	0.001
	(0.001)	(0.001)	(0.001)	(0.001)	(0.001)	(0.001)
Constant	0.574***	0.625***	0.557***	0.688***	0.506***	0.585***
	(0.022)	(0.047)	(0.030)	(0.030)	(0.025)	(0.036)
Year effect	Yes	Yes	Yes	Yes	Yes	Yes
Industry effect	Yes	Yes	Yes	Yes	Yes	Yes
Observation	79029	22190	50211	51010	56642	44579
R-square	0.029	0.067	0.039	0.039	0.019	0.060

Notes: The t-statistic in parenthesis.

***, **, and * indicate statistically significant at 1%, 5%, and 10%, respectively.

The results in columns (1) and (2) of Table 6 indicate that the impact of digital transformation on export product quality is significantly higher in general trade enterprises than in those engaged in processing trade. This is because general trade enterprises are engaged in the complete range of activities along the value chain from R&D to sales. These firms have abundant resources in reserve and are highly capable of transformation, which leads to a greater demand for improvements in product quality. Processing trade enterprises are instead engaged mainly in low value-added activities like processing and product assembly where they are tasked with fulfilling orders. These enterprises have a relatively lower demand to improve production capacity and a limited means to transform, leading to a lower demand for improvements in product quality [2]. Digital transformation therefore has a significant effect on improving the export product quality of general trade enterprises. The results in columns (3) and (4) of Table 6 show that digital transformation also has a more pronounced effect on enterprises with strong corporate governance. One possible reason is that corporate governance can directly reflect a firm’s capacity for technological integration and the allocation of resources. The stronger its capacity for corporate governance, the more a firm is able to integrate technology and resources to improve its export product quality. The results in columns (5) and (6) of Table 6 indicate that digital transformation affects export product quality more significantly in high-tech industries. This is because enterprises engaged in high-tech industries are technology-rich and have a stronger demand for high-end services such as R&D, design, and information, all of which are conducive to the improvement of their export product quality.

*Heterogeneity in the digital transformation of enterprises*. Enterprises vary in their endogenous motivations and capabilities concerning digital transformation. This study divides digital transformation into different levels relative to the average and examines their different effects on export product quality. An enterprise is considered to have a high level of digital transformation if it is above the average, and vice versa. The specific test results are shown in columns (1) and (2) of Table 7, underscoring that higher digital transformation does indeed result in greater improvements in export product quality.

**Table 7:** Results of heterogeneity analysis.

Variables	(1)	(2)	(3)	(4)
	Higher digital transformation	Lower digital transformation	Higher level of digital infrastructure	Lower level of digital infrastructure
Dig	0.031***	0.005	0.010**	0.011
	(0.007)	(0.009)	(0.005)	(0.007)
lnes	0.005***	0.000	0.003**	0.003*
	(0.001)	(0.002)	(0.001)	(0.001)
rrs	0.014	-0.084***	-0.024	-0.062***
	(0.013)	(0.021)	(0.015)	(0.020)
mfr	0.075***	-0.170***	-0.024	-0.021
	(0.017)	(0.026)	(0.019)	(0.024)
tq	0.029***	0.015***	0.019***	0.021***
	(0.003)	(0.003)	(0.002)	(0.004)
lngs	-0.008***	-0.003***	-0.003***	-0.012***
	(0.001)	(0.001)	(0.001)	(0.001)
Constant	0.485***	0.640***	0.556***	0.707***
	(0.032)	(0.042)	(0.024)	(0.039)
Year effect	Yes	Yes	Yes	Yes
Industry effect	Yes	Yes	Yes	Yes
Observation	63289	37931	64510	36711
R-square	0.035	0.039	0.043	0.032

Notes: The t-statistic in parenthesis.

***, **, and * indicate statistically significant at 1%, 5%, and 10%, respectively.

*Heterogeneity at the level of regional digital infrastructure*. Considering that the current level of digital development varies greatly between regions in China, does the impact of digital transformation on a firm’s export product quality also result in heterogeneity? To this end, this paper refers to the work of Pan et al. [82] to construct indicators for the infrastructure of the digital economy and calculates the level of digital infrastructure in each province. A province is considered as having a high level of digital infrastructure if it is above the median level of all provinces and vice versa. The specific test results are shown in columns (3) and (4) of Table 7. They indicate that a higher level of digital infrastructure in a province indeed corresponds to digital transformation having a greater effect on the export product quality of its firms.

## Conclusions and implications

### Conclusions

Harnessing the power of digital technology has become the central driving force for the evolution and growth of different industries in the midst of the digital revolution. Leveraging the dividends of the digital economy to enhance export product quality and become more competitive in foreign trade will be crucial in reshaping the image of Made in China. This paper makes use of the data of listed companies in China from 2000 to 2015 to examine the influence of digital transformation on export product quality. It analyzes the endogenous determinants of export product quality from the theory of enterprise heterogeneity to explore the influence mechanisms of digital transformation. The main conclusions are as follows: First, the digital transformation of an enterprise significantly improves export product quality, and the empirical findings still hold after a series of robustness tests. Second, digital transformation improves export product quality through process productivity (*φ*) and product productivity (*ξ*). Third, the heterogeneity analysis finds that digital transformation affects export product quality the most for firms that are engaged in general trade, those in high-tech industries, and those with a stronger capacity for corporate governance. At the same time, the differences in the surrounding digital infrastructure and level of digital transformation among enterprises also affects the manner in which digital transformation improves quality.

### Policy implications

Based on the above findings, this paper points out the following policy implications concerning how firms may enhance the quality of their export products and help to foster a large trade nation:

First, government should accelerate the digital transformation of enterprises and provide an environment that guarantees its realization. The research in this paper shows that a higher level of digital transformation corresponds to greater improvements in a firm’s export product quality. The government should provide financial and policy support for firms that have already undergone digital transformation to promote further improvements while providing subsidies and underwriting measures for those that have not. This will promote digital transformation and improve the efficiency and capacity for quality production among enterprises, thereby upgrading the quality of their products. In addition, the analysis of the level of digital infrastructure across regions of China shows that this also affects how digital transformation improves export product quality. The government should gather investment and promote the construction of big data centers, 5G, and other forms of digital infrastructure regionally to bolster the digital transformation of enterprises and improve the quality of their export products.

Second, enterprises should seize the opportunity for digital transformation to improve their efficiency and ability to innovate, making their products more competitive in overseas markets. The research in this paper shows that total factor production efficiency and the capacity for technological innovation are the primary means by which digital transformation improves the export product quality of an enterprise. Firms should therefore seize the opportunity provided by digital transformation to reorganize and optimize how innovation is managed, cultivating their ability to absorb and integrate various technical resources. They should also strengthen their use of digital technology in production, management, and sales while investing in artificial intelligence, robotics, interconnection platforms, intelligent sensors, and other intelligent equipment, as doing so will improve precision, efficiency, and the quality of their products.

Third, digital transformation should be targeted at improving the export product quality of different types of firms. Considering that differences in trade modes, technical attributes, and governance capabilities among firms change the way that digital transformation improves their export product quality, both the government and enterprises should take targeted measures to maximize these effects. The government should prioritize financial support to enterprises engaged in general trade and high-tech industries, and enterprises that have already realized digital transformation should strengthen their corporate governance. Low-tech enterprises should strengthen their expenditures in technological R&D and innovation, applying digital transformation to all aspects of their production and operations and seizing the opportunity to improve their export product quality.

## Shortcomings and outlook

First, this paper has measured the export product quality of firms from an industry-wide perspective. As industries are highly varied, future research can further explore the factors influencing export product quality within each industry. Second, further research can explore how export quality is affected in the different dimensions of digital transformation. As there may be differences in the mechanisms through which the various dimensions of digital transformation affect product quality, future research could further explore the impact on product quality at the level of the various dimensions of digital transformation.

## Supporting information

S1 Table The data of all variables. (ZIP)
